# Psychomotor semiology in depression: a standardized clinical psychomotor approach

**DOI:** 10.1186/s12888-022-04086-9

**Published:** 2022-07-15

**Authors:** A. Paquet, A. Lacroix, B. Calvet, M. Girard

**Affiliations:** 1 Department of research and innovation, Centre Hospitalier Esquirol, Limoges, France; 2 INSERM, Univ. Limoges, IRD, U1094 Tropical Neuroepidemiology, Institute of Epidemiology and Tropical Neurology, GEIST, Limoges, France; 3 University Paris-Saclay, UVSQ, Inserm U1018, CESP, Team DevPsy, Villejuif, France

**Keywords:** Major Depression Disorder, Psychomotor Semiology, Muscular Tone, Perceptual-Motor Skill, Body Image

## Abstract

**Background:**

Although psychomotor symptoms are associated with the clinical symptomatology of depression, they are rarely assessed and standardized clinical evaluation tools are lacking. Psychomotor retardation is sometimes assessed through direct patient observations by clinicians or through a clinical observation grid, in the absence of a standardized psychomotor assessment. In this pilot study, we evaluated the feasibility of standardized psychomotor examination of patients with major depressive disorder (MDD) and detailed a psychomotor semiology in these patients.

**Methods:**

We used a standardized psychomotor assessment to examine 25 patients with MDD and 25 age- and sex-matched healthy controls (HC) and compared their psychomotor profiles. Using standardized tests, we assessed muscle tone and posture, gross motor skills, perceptual-motor skills, and body image/organization. Clinical assessments of depressive symptoms (levels of psychomotor retardation, anxiety, and self-esteem) comprised this detailed psychomotor examination.

**Results:**

All participants were examined using the standardized psychomotor assessment. The main results of the psychomotor examination highlighted low body image of MDD participants (*p* < 0.001). Significant differences between groups were found in passive muscle tone, posture, emotional control, jumping, manual dexterity, walking, and praxis. Among these psychomotor variables, body image, passivity, jumping and rhythm scores predicted an MDD diagnosis.

**Conclusions:**

Beyond the psychomotor retardation known to be present in MDD patients, this examination revealed an entire psychomotor symptomatology characterized by elevated muscle tone, poor body image associated with poor self-esteem, slowness in global motor skills and manual praxis, and poor rhythmic adaptation.

In light of these results, we encourage clinicians to consider using a standardized tool to conduct detailed psychomotor examination of patients with depressive disorders.

**Trial registration:**

ClinicalTrials.gov identifier: NCT04031937, 24/07/2019.

**Supplementary Information:**

The online version contains supplementary material available at 10.1186/s12888-022-04086-9.

## Background

Although psychomotor symptoms have received little attention from clinicians, they are a component of major depressive disorder (MDD) symptomatology. Motor behavior, including gross and fine motor activity, discrete body movements, speech, and motor reaction time, can reliably differentiate depressed patients from healthy controls [1, 2]. However, few data and studies are available that could contribute to a thorough understanding of how these symptoms are involved in the general clinical features of depression.

The most common psychomotor characteristics associated with depression are slowing (retardation) or increased activity (agitation). Psychomotor agitation or retardation is a symptomatic and diagnostic criterion of MDD according to the Diagnostic and Statistical Manual of Mental Disorders, 5^th^ edition (DSM-5) [3]. Motor retardation affects all body movements, including gait, facial expressions and verbal output [4]. A depressed person's gait is sluggish and slower, and their posture results in falls and changes in stability when walking [5, 6]. Additionally, spontaneous gestures are reduced [7], facial mobility is diminished,mimicry is poor, and eye contact is avoided [8]. These signs of psychomotor retardation are not necessarily all found in all individuals, but they characterize the overall psychomotor characteristics of a depressed individual [4]. Psychomotor symptoms may serve as predictive criteria of the evolution of pathology or of the therapeutic effect. Notably, they predict an efficient clinical response to tricyclic antidepressants [9, 10], electroconvulsive therapy [11] or repeated transcranial magnetic stimulation (rTMS) treatment [12]. These psychomotor symptoms may also be a vulnerability factor for depression in adolescents [13]. Not all patients with depression exhibit psychomotor retardation and the existence of other specific psychomotor disorders is not known. Thus, exploration is warranted to determine the extent and potential implications of psychomotor symptoms. Knowledge of psychomotor symptoms in depression has been limited by methodological and theoretical developments [9, 10, 14]. Generally, psychomotor function is determined by doctors during clinical observations that entail heteroquestionnaires of varying degrees of specificity that are completed during a medical consultation. Clinical scales, such as the Salpêtrière Retardation Rating Scale [15],CORE [16, 17] or Motor Agitation and Retardation Scale [8], assess psychomotor retardation (body, face, and voice), psychic slowdown and/or psychomotor agitation. These scales are based on behavioral observations of patients but not on tests involving movement that require subjects to physically engage in actions. More objective measures of motor function have been used, but only in experimental situations, in the context of clinical research [9]. Gross motor assessment involves movement patterns, physical performance [18, 19] or accelerometry-based activity monitoring [11] related to the speed, force or kinematics of the movement. Fine motor activity is most often assessed by copying tasks involving writing or drawing [20, 21], and the actigraphy provided measurements of body movements during daily life via a portable device [11, 22]. These evaluation procedures are essentially based on a quantitative analysis of the movement (reaction time, gait speed, stride length, and maximal force) and most often highlight psychomotor slowing, but the qualitative aspects of movement (position, posture, and quality of gesture) are not reported. Moreover, these measures are not used in clinical practice to diagnose or monitorthe disease.

Thus, specific standardized tools for assessing psychomotor function are lacking. In practice, the evaluation of psychomotor symptomatology consists of a rapid and imprecise assessment by clinicians on the basis of their own observations or from the scales previously mentioned.

However, these assessments fail to reveal psychomotor symptom profiles, including minor deficits (neurological soft signs, NSSs) that are impossible to detect without a psychomotor examination based on specific tests. NSSs correspond to discrete and diffuse neurological abnormalities characterized by a lack of sensory integration, motor coordination disorders, lateralization disorders, or abnormal movements. Unfortunately, there are no data concerning the existence or absence of NSSs in depression, although several studies have reported the presence of such signs in other psychiatric pathologies, such as schizophrenia or bipolar disorder [23, 24]. Thus, to the best of our knowledge, there are no references or studies on motor coordination, muscle tone, or sensorimotor integration in patients with depression.

A psychomotor examination, of which psychomotor therapists are specialists, is a tool that allows the psychomotor profile of a subject to be characterized from a perceptual-motor approach. This examination is based on targeted investigations, using standardized tools, to evaluate the qualitative and quantitative aspects of movement and to understand the subject's body representations. In this study, we aimed to evaluate several psychomotor functions in patients with MDD based on a standardized psychomotor examination. A detailed exploration of psychomotor function could help characterize the psychomotor semiology in depression.

## Methods

### Aims and design of the pilot study

Our main objective was to compare the results of psychomotor tests between MDD participants and healthy subjects. First, we conducted a standardized psychomotor assessment of 25 patients with MDD and 25 age- and sex-matched HCs, and compared their psychomotor profiles.

Second, we investigated the relationships between clinical and psychomotor variables, as well as the potential predictive value of psychomotor variables for the presence or absence of MDD.

### Participant description

MDD participants were recruited at the Esquirol Hospital (Limoges, France) during consultations or during hospitalization for MDD. All were diagnosed with MDD by a psychiatrist according to the DSM-5. MDD patients with psychiatric comorbidities (bipolar disorder, addictions other than tobacco, eating disorders, or obsessive–compulsive disorders) and, those undergoing neuroleptic treatment and/or undergoing electroconvulsive therapy were excluded.

Healthy controls (HCs) were recruited by posters placed in the Esquirol Hospital's staff rooms and by word of mouth. The inclusion criteria for HCs were validated by a clinician. Only people without a history of mental illness; current or previous depressive disorder; or treatment with antidepressants, neuroleptics, anxiolytics and/or hypnotics (benzodiazepines) were included.

In both groups (MDD and HC), participants were between 18 and 65 years of age, without a known neurological history or impairment, neurodevelopmental disorder, or sensory or physical disability, and were able to answer questionnaires and understand French; moreover, they were not recruited from population protected by French law.

Participants in the two groups were matched for age and sex. Each groups was comprised of 17 women and 8 men. The average age of MDD participants was 42.9 ± 12.7 years and that of HC participants was 43.5 ± 11.5 years (Additional file 1).

The study was conducted from October 2018 to October 2019. It was approved by an ethics committee (French committee for the protection of individuals- Ile de France V) as required by French regulations (Clinical Trials number NCT04031937, 24/07/2019), and written informed consent was obtained from all participants. The study was carried out in accordance with the principles of the Declaration of Helsinki.

### Psychometric assessments

- The Hamilton Depression Rating Scale 17-item version (HDRS-17) is used to characterize the intensity of depressive symptoms [25, 26]. The threshold scores for depression are as follows: < 7, no depression; 8 to 16, mild depression; 17 to 23, moderate depression; and > 24, severe depression [27]. Items 7 (work and activity), 8 (slowdown), and 9 (agitation) specifically relate to motor function.
- The Hamilton Anxiety Rating Scale (HARS) includes 14 items that assess psychic, somatic, muscular, and visceral anxiety, cognitive and sleep disorders, and depressed mood, thus evaluating the intensity of anxiety [28].
- The Clinical Outcomes in Routine Evaluation (CORE) was used to quantify the degree of psychomotor retardation [7]. This scale is based on an observational analysis during an interview. The CORE score quantifies the degree of psychomotor impairment and can be used to identify patients suffering from melancholic depression. The cutoff score used to characterize melancholic depression was 8 points [29].
- The Rosenberg Self-Esteem Inventory (RSEI), which consists of a self- administered questionnaire containing 10 items was used to measure subjects' self-esteem [30].

### Psychomotor examination

**Fig. 1:**
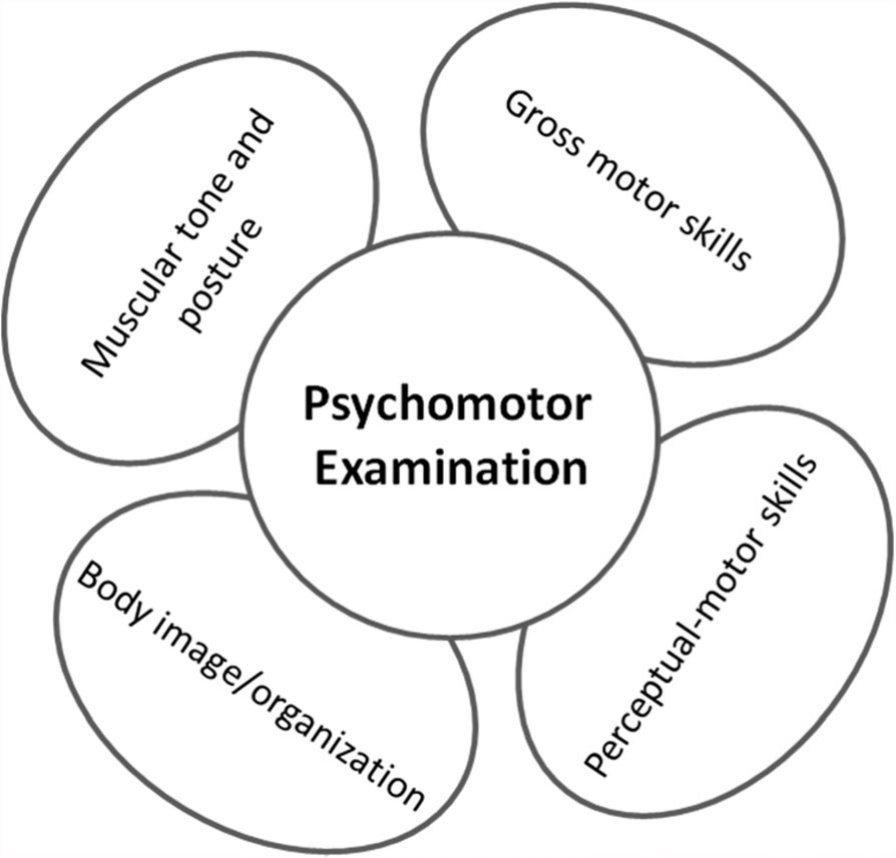
Four domains assessed by the psychomotor examination

The psychomotor examination assessed four domains (muscle tone and posture, gross motor skills, perceptual-motor skills, and body image/organization) (Fig. 1) and, involve several tasks from different standardized tools.

Many of these tasks were derived from a French psychomotor (NP-MOT) battery [31] that provides a developmental assessment of neuropsychomotor integration. Although generally used with children, the tests used can be applied to adults because of the developmental nature of the tasks. The NP-MOT battery allows a discriminant analysis of neuropsychomotor and sensory-motor functions to better understand the nature of psychomotor disturbances. The NP-MOT battery explores various functions with one or more tests each, some of which consist of several items. The assessment method is listed in Table 1. This battery is a standardized instrument to assess qualitative (gesture achievement) and quantitative (speed) aspects of movements for each of the functions explored. The NP-MOT tasks have been previously described by Paquet et al. [32, 33].

- Muscle tone and posture assessments included evaluations of passive and active Muscle tone (NP-MOT battery) and expressive tone from tonic-emotional reactions (e.g., jerky breathing, vasomotricity, sweating, and tremors). CORE items related to posture or body expressiveness were analyzed separately in this section (item 2: facial immobility, item 3: posture, item 10: corporal immobility, item 11: motor agitation, item 13: slowness of movements, and item 15: delay in motor activity).
- The gross motor skill assessment included dynamic and static balance as well as manual dexterity (NP-MOT battery).
- The perceptual-motor skill assessment included manual praxis (bimanual, manual and fingers), body spatial integration, rhythm, finger tactile gnosis (NP-MOT battery), and visuospatial-motor structuration evaluation by the Rey complex figure test (RCF copy only) [34].
- The body image/organization assessment included body image from the Body Image Questionnaire (BIQ) [35], a scale measuring body satisfaction. Body organization was assessed with a French (CORP-R) battery [36, 37] that evaluates subjects' representation of their own bodies and of the relationships between the different parts of their body through the manipulation of nonjoining puzzle pieces. The test included two levels of representation of the body and the face: full-face and from the side.

**Table 1:** Tasks from the French neuropsychomotor (NP-MOT) battery

Functions		*Items*	*Observation*
**Muscular tone**	Passive muscle tone	*Arm dropping test*	*Arm fall quality (fall, brake, or no fall)*
		*Wrists dangling*	*Angles and resistance between both wrists*
		*Extensibility of wrists*	*Angles and resistance between both wrists*
		*Extensibility of shoulders*	*Joint resistance and stretching of the shoulder*
		*Passivity*	*Search for muscle rigidity during arm manipulation*
		*Anklesdangling*	*Angles and resistance between both ankles*
		*Extensibility of the ankle, legs stretched or bent*	*Angles and resistance between both ankles*
		*Motor pathway disorder (mild sign)*	*Presence or lack of distal spasticity*
		*Intentional muscular relaxation*	*Search for muscle rigidity on passive mobilization of the upper and lower limbs, lying down*
	Active muscle tone	*Diadochokinesis*	*Movement quality and synkinetic movements during prono-supination*
	Expressive tone	*Emotional control*	*Tonic-emotional reactions (e.g., jerky breathing, vasomotricity, sweating, tremors, *etc*.)*
**Gross motor skills**	Dynamic balance	*Walk a line forward/backward*	*Posture: upper limbs and lower limbs*
		*Walk on tiptoe*	*Tiptoe position*
		*Walk on heels*	*Heel position*
		*Jumping*	*Coordination and landing*
	Static balance	*Feet together, eyes closed, outstretched arms*	*Feet position, arm deviation*
		*On one foot*	*Arm deviation and duration*
		*On tiptoe*	*Arm deviation and duration*
**Manual dexterity**		*Grab tokens quickly*	*Gesture quality and duration*
**Manual praxis**		*Symmetrical and asymmetrical bimanual praxis*	*Gesture quality and duration during prono-supination*
		*Thumb/index opposition: right/left*	*Gesture quality, duration and synkinesis*
		*Ideomotor gnosopraxis (manual)*	*Realization of gesture imitation with hands*
		*Ideomotor gnosopraxis (digital)*	*Realization of gesture imitation with fingers*
**Body spatial integration **		*Bodily spatial integration to self*	*Knowledge of right and left by pointing and/or verbal command for an axial crossing gesture*
		*Bodily spatial integration to other*	
		*Bodily spatial integration to object*	*Knowledge of right and left from objects*
**Rhythm**		*Tapping*	*Spontaneous speed of regular hand taps on the table*
	Auditory-perceptual-motor task (APM tasks)	*Clap hands at the same time as a metronome* *Walk at the same time as a metronome*	*Hand clapping adaptation to match the rate set by a metronome* *Walking adaptation to match the rate set by a metronome*
**Finger perception**		*Finger tactile gnosis*	*Localization of fingers to tactile stimuli on the concealed hand*

Executive functions were assessed with the Frontal Assessment Battery (FAB) [38].

### Procedure

Patients with MDD and HCs were individually assessed by a psychomotor therapist trained on the tests. The tests for both groups were carried out in the same order: collection of sociodemographic and treatment data and completion of the HARS, RSEI, FAB, BIQ and psychomotor tasks. Due to ethical considerations, the HDRS and CORE were carried out by MDD participants only (see Additional file 1). Assessments were not blinded to patient status. Indeed, they were made using self-administered questionnaires or objective measures from standardized tests to limit the influence of the examiner on the participants' results.

### Statistics

Statistical analyses were conducted using SPSS® version 22 (Statistical Package for Social Science) commercial software for Windows on a PC. Qualitative variables are presented as percentages and numbers; quantitative variables are presented as the means and standard deviations. Comparisons between groups on categorical variables were performed using Fischer’s test, and the means of quantitative variables were compared between groups using the nonparametric Mann–Whitney test. Bonferroni correction were applied to control for multiple comparisons related to CORE items (*p* = 0.008), walk score (*p* = 0.007), static balance score (0.0125), and rhythm score (*p* = 0.017) due to the interdependence of the variables.

Spearman correlation analyses were used to examine the relationships between depressive symptomatology (global scores on the HDRS, HARS, and CORE; and items 7- 9 of the HDRS) and psychomotor functions that differed between patients with MDD and HCs. To identify significant predictive psychomotor factor(s) for MDD, we performed binary logistic regressions with stepwise forward selection of variables and with the group (MDD or HC) as the dependent variable. We evaluated several models corresponding to each psychomotor field of interest: muscle tone and posture, gross motor skills, perceptual-motor skills and body image/organization. The explanatory variables from these fields that exhibited group differences were entered into the specific regression model and filtered according to the following criteria: variables with *p* < 0.2 and not correlated with each other.. The regression model using body image variables included only the BIQ score.

For each statistical analysis, the significance threshold was 5%.

## Results

### Description of clinical characteristics

Table 2 shows the data collected from both groups, which were were similar in age and sex by design. No participant in the HC group received psychotropic treatment. The mean HDRS score of MDD participants was 19.6 ± 5.3 [16.5–23.5], indicating moderate depression. The CORE score for patients with MDD was 4 ± 4.2 [0.5–6.5]. Five patients had a CORE score > 8, suggesting melancholic symptoms. Patients with MDD were significantly more anxious (HARS scores) and had lower self-esteem (RSEI scores) than participants in the HC group. No difference in executive function (FAB scores) was observed between the two groups. All participants could be examined using the standardized psychomotor assessment.

**Table 2:** General characteristics of the MDD and HC groups

	MDD (*n* = 25)	HC (*n* = 25)	*P* value
Age (mean ± SD)	42.9 ± 12.7	43.5 ± 11.5	0.82
Sex ratio (M/F)	8/17	8/17	1
Treatment (n)			
*Antidepressants*	21	-	-
*Anxiolytics*	23	-	-
*Hypnotics*	7	-	-
*rTMS*	1	-	-
HDRS-17 score (mean ± SD)	19.6 ± 5.3	-	-
CORE score (mean ± SD)	4.0 ± 4.2	-	-
HARS score (mean ± SD)	20.1 ± 5.8	4.4 ± 4.6	< 0.001
FAB score (mean ± SD)	16.6 ± 1.1	17 ± 0.9	0.27
RSEI score (mean ± SD)	21.0 ± 5.0	33.3 ± 3.8	< 0.001

### Comparison of MDD patients and HCs on psychomotor performance

Overall, the HC group performed better, on psychomotor tasks than the MDD group (Tables 3, 4 and 5). In the evaluation of muscle tone and posture, scores on the two tasks that assessed passive muscle tone (*arm dropping* and *passivity*), the emotional control assessment and the related CORE item significantly differed between the MDD and HC groups (Table 3).

**Table 3:** Results on the muscle tone and posture tasks (% success; mean (standard deviation))

	**Task (battery)**	**MDD** **(*n* = 25)**	**HC** **(*n* = 25)**	***p* value**	
Muscle tone and posture	**Passive muscle tone (NP-MOT)**				
	→ Arm dropping test %	36	72	0.022*	(F)
	→ Wrists dangling	4.00 (0.00)	4.00 (0.00)	1	(MW)
	→ Extensibility of wrists	4.00 (0.00)	4.00 (0.00)	1	(MW)
	→ Extensibility of shoulders	5.2 (1.04)	5.32 (0.94)	0.352	(MW)
	→ Passivity %	32	68	0.023*	(F)
	→ Ankles dangling	3.88 (0.44)	3.84 (0.55)	0.542	(MW)
	→ Extensibility of the ankles	3.88 (0.44)	3.76 (0.66)	0.116	(MW)
	→ Motor pathway disorder (mild sign)%	4	0	1	(F)
	→ Intentional muscular relaxation	3.16 (1.14)	3.64 (0.76)	0.072	(MW)
	**Active muscle tone (NP-MOT)**				
	→ Dysdiadochokinesis	3.96 (0.20)	3.76 (0.66)	0.156	(MW)
	→ Synkinetic movements	22.84 (1.21)	23 (1.96)	0.152	(MW)
	**Expressive tone**				
	→ Emotional control %	100	80	0.025*	(F)
	**Posture (CORE)**				
	→ Item 2 (facial immobility) ^*a*^	0.48 (0.71)	0 (0)	0.001*	(MW)
	→ Item 3 (posture) ^*a*^	0.16 (0.37)	0 (0)	0.039	(MW)
	→ Item 10 (corporal immobility) ^*a*^	0.20 (0.41)	0 (0)	0.020	(MW)
	→ Item 11 (motor agitation) ^*a*^	0.08 (0.28)	0 (0)	0.153	(MW)
	→ Item 13 (slowness of movements) ^*a*^	0.32 (0.75)	0 (0)	0.020	(MW)
	→ Item 15 (delay in motor activity) ^*a*^	0.24 (0.52)	0 (0)	0.020	(MW)

*F* Fischer test, *MW* Mann–Whitney test

^*a*^Bonferroni correction

^*^Significant results

**Table 4:** Results on the gross motor skills tasks from the NP-MOT battery (mean (standard deviation))

	***Task***	***MDD*** ***(n* = *25)***	***HC*** ***(n* = *25)***	***P value***
**Gross motor skills**	**Dynamic balance**			
	→ *Walk: total score *^*a*^	24.94 (1.94)	25.48 (1.73)	0.045
	→ *Walk a line forward: total score *^*a*^	5.32 (0.48)	5.6 (0.50)	0.049
	→ *Walk a line forward: time score *^*a*^	2.32 (0.47)	2.6 (0.5)	0.049
	→ *Walk a line forward: posture score *^*a*^	3.00 (0.00)	3.00 (0.00)	1
	→ *Walk a line backward: total score *^*a*^	4.36 (1.44)	4.94 (1.41)	0.075
	→ *Walk a line backward: time score *^*a*^	1.96 (0.79)	2.52 (0.59)	0.009*
	→ *Walk a line backward: posture score *^*a*^	2.4 (1.22)	2.4 (1.22)	1
	→ *Walk on heels*	3.76 (0.66)	3.96 (0.20)	0.156
	→ *Walk on tiptoe*	4.00 (0.00)	4.00 (0.00)	1
	→ *Jump*	7.52 (0.93)	7.88 (0.33)	0.007*
	**Manual dexterity**			
	→ *Manual dexterity*	10.24 (2.30)	11.52 (0.92)	0.010*
	**Static balance**			
	→ *Static balance: total score *^*a*^	15.72 (1.84)	16.44 (1.00)	0.126
	→ *Feet together *^*a*^	4.72 (1.06)	5.00 (0.00)	0.153
	→ *On one foot *^*a*^	7.40 (0.87)	7.68 (0.63)	0.263
	→ *On tiptoe *^*a*^	3.60 (0.76)	3.76 (0.60)	0.341

^*a*^Bonferroni correction

^*^Significant results

**Table 5:** Results on the perceptual-motor skills tasks (mean (standard deviation))

	***Task (battery)***	***MDD*** ***(n* = *25)***	***HC*** ***(n* = *25)***	***p value***
**Perceptual-motor skills**	**Manual praxis (NP-MOT)**			
	→ *Symmetrical bimanual praxis*	6.64 (0.57)	6.84 (0.37)	0.174
	→ *Asymmetrical bimanual praxis*	6.20 (0.96)	6.68 (0.80)	0.017*
	→ *Thumb/fingers opposition*	13.80 (0.41)	13.92 (0.40)	0.102
	→ *Ideomotor gnosopraxis (manual)*	9.86 (0.44)	10.00 (0.00)	0.077
	→ *Ideomotor gnosopraxis (digitals)*	15.38 (0.63)	15.16 (1.02)	0.650
	**Body spatial integration (NP-MOT)**			
	* → Body spatial integration: total score*	16.32 (2.58)	17.04 (1.43)	0.531
	**Rhythmic task (NP-MOT)**			
	→ *APM task: total score *^*a*^	10.96 (1.59)	11.84 (0.37)	0.048
	→ *Clap hands (APM task) *^*a*^	5.64 (1.04)	5.88 (0.33)	0.877
	→ *Walk (APM task) *^*a*^	5.32 (1.21)	5.96 (0.20)	0.018
	→ *Tapping*	5.68 (1.6)	6.20 (1.15)	0.186
	**Finger perception (tactile gnosis NP-MOT)**	19.52 (1.26)	19.80 (0.41)	0.638
	**Visuospatial-motor structuration (RCF)**			
	→ *Rey complex figure test: copy*	65.84 (4.38)	66.88 (3.32)	0.404
	→ *Rey complex figure test: time*	170.76 (78.34)	129.56 (48.19)	0.150

^*a*^Bonferroni correction

^*^Significant results

Concerning gross motor skills, performance on the dynamic balance tasks (*time score for walking backward in a line* and *jumping score)* and the *manual dexterity task* significantly differed between the MDD and HC groups (Table 4). When examining balance, we found that one MDD participant failed the feet-together test due to a slow and progressive lateral tilt after occlusion of the eyes, suggesting a labyrinthine Romberg sign. The identification of this postural control deficit, which may be of vestibular origin, led the medical staff to issues a referral for a neurological examination.

Among the perceptual-motor skills, performance on one manual praxis task (*asymmetrical bimanual praxi*s) significantly differed between MDD and HC groups (Table 5), and one rhythm task (*walk task of the APM test*) exhibited a tendency to differ.

Regarding body image/organization, the BIQ scale results significantly differed between the MDD and HC groups (52.24 ± 12.31 and 75.04 ± 7.86, respectively, *p* < 0.0001). The HCs had a better body image than the MDD participants. Body organization did not differ between the MDD and HC groups for either the *CORP-R full-face test* (32.48 ± 0.77 versus 32.32 ± 1.25, respectively; *p* = 0.856) or the *CORP-R from the side test* (31.60 ± 3.30 and 31.64 ± 3.24, respectively; *p* = 0.784).

### Correlations between depressive symptomatology and significant psychomotor variables

A strong correlation between *BIQ score* and self-esteem (*r* = 778; *p* < 0.001) was found, supporting the link between low self-esteem and poor body image. Two variables were significantly correlated with the HARS score: the *BIQ score* (*r* = -0.40, *p* = 0.049) and the *APM task total score* (*r* = 0.40, *p* = 0.047). The more anxious the MDD participants were, the lower their body image was and the more difficulty they experienced adapting to the rhythm. *Emotional control* was negatively correlated with activity and work items on the HDRS(*r* = -0.45, *p* = 0.023), and the *time score for walking backward in a line* was negatively correlated with items of the HDRS that evaluated slowing (*r* = -0.50, *p* = 0.012).

### Psychomotor factors predictive of an MDD diagnosis

First, we conducted a binary logistic regression to identify muscle tone and posture variables (*passivity*, *extensibility of the foot*, *dysdiadochokinesis*, *synkinetic movements*, *emotional control*, *CORE item 2*, and *CORE item 11)* that predicted an MDD diagnosis.. The results were statistically significant, indicating that muscle tone and posture significantly predicted group (MDD or HC) (χ^2^ = 27.55, *p* < 0.001, df = 7) and explained 56% of the variance in an individual’s likelihood of having MDD (Nagelkerke’s R^2^ = 0.56). After these variables were introduced into a stepwise linear regression (Table 6 a), the *CORE Item 2 score* along with the *passivity score* and *emotional control score* accounted for 53% of the variance in MDD diagnosis.

**Table 6:** Predictive factors (in the stepwise binary logistic regression) of MDD diagnosis from tasks assessing muscle tone and posture (a), gross motor skills (b), and perceptual-motor skills (c)

	β	p	OR (95% CI)
a			
Constant	-1.45	0.009	0.23
* Passivity*	1.56	0.034	4.78 (1.12, 20.32)
* Emotional control*	21.13	0.999	1.51E^+^9 (0.00, -)
* CORE Item 2 (facial immobility)*	20.28	0.998	6.43E^+^8 (0.00, -)
Note: global % = 76, χ^2^ = 25.356, R^2^ Nagelkerke = 0.53, ddl = 3, *p* < 0.001			
Β: regression coefficient, OR: odds ratio, 95% CI: 95% confidence interval			
b			
Constant	25.82	0.006	1.63E^+^11
* Jumping*	-1.84	0.052	0.16 (0.02, 1.01)
* Manual dexterity*	-0.72	0.011	0.487 (0.28, 0.85)
* Walk a line backward: time score*	-1.66	0.014	0.190 (0.05, 0.72)
Note: global % = 78, χ^2^ = 25.125, R^2^ Nagelkerke = 0.53, ddl = 3, *p* < 0.001			
Β: regression coefficient, OR: odds ratio, 95% CI: 95% confidence interval			
c			
Constant	10.48	0.040	3.57^E+^4
* APM task: total score*	-0.91	0.037	0.40 (0.17, 0.95)
Note: global % = 60, χ^2^ = 7.915, R^2^ Nagelkerke = 0.195, ddl = 1, *p* = 0.005			
Β: regression coefficient, OR: odds ratio, 95% CI: 95% confidence interval			

For the regression relative to gross motor skills, the scores included: the *time score for walking forward in a line, time score for walking backward in a line, jumping score*, *manual dexterity score*, *and the score for walking on one’s heels*. The model was statistically significant, indicating that gross motor skill variables significantly predicted group (χ = 25.66, *p* < 0.001, df = 5) and explained 53% of the variance in an individual’s likelihood of having MDD (Nagelkerke’s R^2^ = 0.53). After these variables were introduced into a stepwise linear regression (Table 6 b), the *jumping score* along with the *manual dexterity score* and the *time score for walking backward in a line* accounted for 53% of the variance in MDD diagnosis.

For the regression relative to perceptual-motor skills, the scores included: *bimanual praxis asymmetric, thumb/finger opposition, ideomotor gnosopraxis (manual), APM task total*, *tapping and Rey complex figure test: time.* The model was statistically significant, indicating that perceptual-motor skills significantly predicted the two groups (χ = 23.28, *p* < 0.001, df = 6) and explained 50% of the variance in an individual’s likelihood of having MDD affected a person’s risk of belonging to the MDD group by 50% (Nagelkerke’s R^2^ = 0.50). After these variables were introduced into a stepwise linear regression (Table 6 c), the *APM task total score* predicted MDD diagnosis. This variable accounted for 19% of the variance in MDD diagnosis.

Concerning body image/organization, the *BIQ score* predicted group membership with high significance (*p* < 0.0001). The *BIQ score* affected the risk of belonging to the MDD group by 71% (β = -0.21; odds ratio (OR) = 0.81, 95% confidence interval (CI) = 0.72, 0.91).

## Discussion

In this study, we compared the psychomotor functions of adults with MDD and adults without psychiatric disorders to describe and identify the potential MDD psychomotor profile. We demonstrated the use of a standardized psychomotor examination in MDD participants. We also observed significant differences in performance between MDD and HCs on several psychomotor tasks assessing muscle tone and posture (*arm dropping test*; *passivity; emotional control; and CORE items 2*), gross motor skills (*walking backward a line: time score, jumping score, and manual dexterity score*), perceptual-motor skills (*asymmetrical bimanual*) and body image/organization (*BIQ score*). Patients with MDD performed worse on these tasks.

There were no group differences in physiological muscle tone (*extensibility, dangling and no motor pathway disorder*) or active muscler tone (*synkinesia)* on the muscle tone and posture tasks. However, the MDD and HC groups differed in passive muscle tone. Passivity during limbs mobilizations (*passivity and intentional muscular relaxation*) was challenging for participants with MDD. These individuals exhibited more paratonia and more tonic-emotional reactions (*emotional control*), e.g., jerky breathing and vasomotricity, during passive muscle tone tests. This difficulty in voluntarily achieving muscle relaxation was obvious on the *arm dropping test*, which most participants with MDD failed. The high level of tonic-emotional reactivity observed in participants with MDD contrasted with the apparent motor “passivity” evidenced by score on the selected CORE items, which were characterized by hypoactivity, immobility, lack and slowness of movement or delay in activity. Thus, an apparent psychomotor delay or slowness may mask high body tension, which can only be assessed by a clinical examination of muscle tone. Muscle tone results indicated tonic-emotional dysregulation with corporal manifestations in participants with MDD, suggesting lower emotional control abilities compared with controls. Notably, MDD is characterized by abnormalities in emotion regulation [39] and dysregulation of major biological stress systems [40],which impact personal and social aspects of lives. Both abnormalities can affect muscler tone by tonic-emotional manifestations. More generally, in human development, each emotion (anxiety or anguish) is accompanied by a tonic [41] or neurovegetative response. This interrelation between muscle tone and emotion is explained by the regulation by shared spinal and cerebral structures. A core network of cortical (prefrontal) and subcortical (various brainstem nuclei, hypothalamus, amygdala, basal ganglia, and anterior cingulate) regions involved in emotion regulation (for a meta-analysis see [42]) also participates in the regulation of muscletone. Moreover, the brainstem, in particular, is involved in the affective component of emotional responses [43]. Subcortical structures send descending projections to brainstem regions and ascending projections to cortical regions that produce the expression and affective components of the emotional response. The reticular formation in the brainstem, influenced by the basal ganglia, affects posture and muscle tone along descending projections [44, 45]. The nerve signals generated by these anatomical structures combine to deliver a message with somatic and psychic origins. Therefore, muscle tone, considered an emotional backdrop [41, 46], exhibits bodily manifestations of affective, emotional, and identity-related phenomena.

Given this interrelation between the body and a person’s affect, a psychomotor evaluation allows us to consider the "tonic dialog" [47]. The significant differences in muscle tone between the two groups and the predictives value of several muscle tone variables (passivity, emotional control, and facial immobility) for depression suggest that assessment of muscle tone indicates the states of bodily and emotional tension and should be incorporated in clinical examinations of individuals with depression.

We also found many group differences in gross motor skills, with MDD participants achieving low scores on dynamic balance (walking tests) and exhibiting major difficulties in speed of execution. This slowness in participants with MDD was also found on the manual dexterity tasks and asymmetrical bimanual praxis tasks. These results are consistent with the slowness of movement among individuals with depression described in the literature [2]. Qualitative analysis of the jumping task showed that MDD participants had more stepover jumps and less or no coordination of their arms compared to HCs, who exhibited better performance in the feet-together task for jumping and better coordination of their arms. Regarding perceptual-motor skills, MDD patients failed the asymmetrical bimanual praxis tasks, which involve coordination linked to functional efficiency of the corpus callosum, more than HCs. Contrary to the findings of Pavlidou et al. [48], we did not find significant group differences in praxis, but we did find a tendency to toward lower scores on manual gnosopraxis. The MDD and HC groups differed only on the rhythmic task. In this task, participants had to clap or walk to the rhythm set by a metronome; theMDD participants had more difficulty walking in rhythm. However, the detailed cognitive profiles of patients were unavailable in this study, and some cognitive impairments could explain their poor performance on perceptual-motor tasks.

Overall, our results showed that MDD participants were slower when performingpsychomotor tasks and that their motor participation (in the jumping and APM tasks) was weaker. Although the use of actigraphy in depression research allows for the identification of motor slowing, sometimes correlated with scales [11, 49], this method, which is rarely used clinically, does not provide objective qualitative measurements aspects of body movement and is a fragile measure of psychomotor agitation. In the present study, the clinical psychomotor approach allowed qualitative and quantitative analysis of performance. This analysis indicated that MDD did not alter global coordination but was associated with differences in execution speed and motor adaptation. The clinical psychomotor approach, with a standardized evaluation, allowed determination of specific motor and psychomotor disturbances and facilitate hypotheses as to the mechanisms involved. Quantitative assessment lacking actigraphy or instrumental measures can only screen for motor slowing or agitation without determining motor disorders, or their underlying mechanisms.

In a recent experimental study based on quantitative movement analysis, Sandmeir et al. (2021) showed that the more severe a patient’s depression was, the less body movement they exhibited. In addition, the authors found that the observed reduction in body movements was not correlated with items assessing motor symptoms on two depression scales (the HDRS and Beck Depression Inventory-II). Similarly, we found that only two psychomotor variables were related to items on the HDRS assessing motor symptoms; furthermore, the psychomotor variables were not correlated with the CORE scores of MDD patients. Other studies also failed to find a correlation between depression scales and instrumental assessments [49, 50], suggesting that the use of scales alone is insufficient for assessing psychomotor disorders.

Low body image, particularly in relation to low self-esteem, was identified in the MDD group. Apart from eating disorders, in which body image has been extensively studied, body image dissatisfaction is often found in patients with physical diseases or injuries and predicts or is associated with psychological distress (depression, anxiety, and self-esteem) or lower quality of life [51–53]. Any change in the body, damage or loss of function alters body image and leads to anxiety [54]. Conversely, the effect of affective disorders, such as depression or anxiety, on body image has received little attentionthus, body image in depression merits further study.

Our study showed that standardized tools specific to psychomotor examination allowed us to highlight significant differences in each of the psychomotor domains studied (muscle tone and posture, gross motor skills, perceptual-motor skills, and body image/organization) between subjects with MDD and healthy subjects. We did not highlight NSSs among patients with MDD; however, the psychomotor examination led to a neurological examination recommendation for one subject.

Several psychomotor variables were identified to predict an MDD diagnosis and merit additional investigation: body image, passive muscle tone and emotional control, gross motor skills (movement quality and speed of execution), and rhythm. To the best of our knowledge, this study is the firstto utilize standardized clinical tools specific to psychomotor functions. We demonstrated the feasibility and value of such an examination. Like Schrijvers et al. [9], we encourage the use of these specific clinical tools in a psychomotor examination carried out by trained professionals (such as psychomotor therapists and occupational therapists). Such assessments help to refine the psychomotor profile of patients, provide information on the semiology of psychomotor disorders, and provide additional psychophysiologicalinformation about depressive symptomatology.

The evaluation outlined in this study allows us to refine the psychomotor profile of patients by identifying possible disorders (psychomotor disorders and NSSs) or difficulties that may impact their daily lives. It also enables further investigation of the psychomotor semiology of depression and enabling the development of psychophysiopathological hypotheses for depressive symptoms, particularly tonic-emotional disorders. The psychomotor profile produced by the psychomotor examination should also allow clinicians to propose adapted no-drug interventions (adapted physical activity, relaxation, mindfulness, and psychomotor therapy) specific to the physical and psychomotor capacities and challenges of patient.

### Limitations

The subtest from the NP-MOT battery used in this study are based on developmental psychomotor tasks that are not influenced by language or culture. However, a validation study on adults from different cultures would be of high importance given the lack of adult developmental norms. Small sample size, especially given the numbers of variables explored, was a limitation. In the future, this psychomotor examination should be conducted with a larger sample, controlling treatment and cognitive affection, to confirm the results of this study.

### Expansions

We did not find any evidence of cognitive impairment in the study participants, although cognitive impairment may be associated with depressive disorders [55, 56]. We were therefore unable to compare the psychomotor profiles of participants with and without cognitive impairment. However, only five out of 25 participants with MDD were identified as exhibiting significant psychomotor retardation according to the CORE scale. In a future study, it would be interesting to include MDD participants with associated cognitive impairment and/or psychomotor retardation, as identified by clinicians to determine if psychomotor profiles differ according to these clinical characteristics.

## Conclusion

Psychomotor examination using standardized tools specific to psychomotor function is relevant to the evaluation of depression. In this study, we identified unique psychomotor characteristics of MDD participants (body image, passive rmuscle tone, emotional control, gross motor skills and rhythm) and performed a semiological analysis. In light of these results, we encourage similar clinical examinations to refine the psychomotor semiology of patients and provide additional psychophysiopathological information about depression. In general, this psychomotor approach may be particularly interesting in the research domain criteria (RDoC) framework, which recently included a sensorimotor domain [57, 58]. Psychomotor impairment associated with depression influences the daily behaviors of patients and should therefore be given more attention, especially as adapted therapeutic targets might improve tpatients’quality of life.

## Supplementary Information

**Additional file 1.** Study design.

## Data Availability

Data are available if required by reviewers at aude.paquet@ch-esquirol-limoges.fr; methods were presented in manuscript and additional file.
