# Supplementary figures and images for: Psychomotor semiology in depression: a standardized clinical psychomotor approach
Source: BMC Psychiatry. 2022 Jul 15;22:474. doi: 10.1186/s12888-022-04086-9 (PMC9287955; doi:10.1186/s12888-022-04086-9)

Additional file 1

Study design


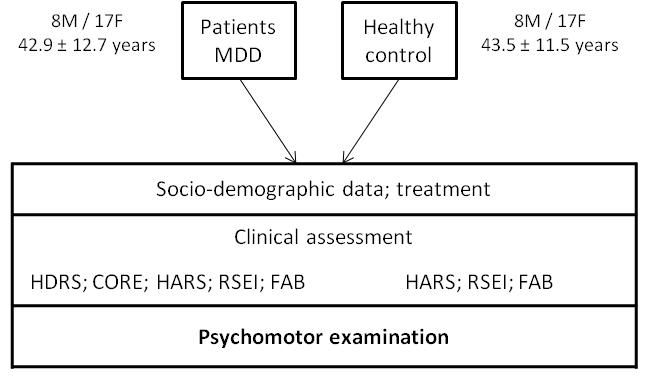

Supplement: Supplementary file 1 — Additional file 1. Study design. [file 12888_2022_4086_MOESM1_ESM.docx]
